# Participatory approaches, local stakeholders and cultural relevance facilitate an impactful community-based project in Uganda

**DOI:** 10.1093/heapro/daz127

**Published:** 2020-02-18

**Authors:** James O’Donovan, Andrew Thompson, Christina Stiles, Japheth A Opintan, Ken Kabali, Ian Willis, Mwebe Edward Mutimba, Elizabeth Nalweyiso, Henry Mugabi, David P Kateete, Matthew Ameniko, George Govina, Rachel Weberman, Edward O’Neil, Niall Winters, Ankur Mutreja

**Affiliations:** 1 Department of Education, University of Oxford, Oxford, UK; 2 Division of Research & Global Health Equity, Omni Med, Mukono, Mukono District, Uganda; 3 Department of Medicine, Addenbrookes Hospital, University of Cambridge, Cambridge, UK; 4 Pritzker School of Medicine, University of Chicago, Chicago, IL, USA; 5 Department of Medical Microbiology, University of Ghana, Korle-Bu, Ghana; 6 Division of Community Services, Omni Med, Mukono, Mukono District, Uganda; 7 Makerere University School of Health Sciences, Makerere University, Kampala, Uganda; 8 Community Health Water and Sanitation Agency, Accra, Ghana; 9 University of Illinois College of Medicine-Peoria, Peoria, IL, USA; 10 Department of Emergency Medicine, St. Elizabeth’s Medical Center, Boston, MA, USA

**Keywords:** community, sanitation, hygiene, Africa, workshop

## Abstract

Sanitation is a major global challenge that is often addressed at national and international levels, while community opinions and beliefs are neglected. To promote water, sanitation and hygiene (WASH) we organized a cross-cultural knowledge exchange workshop to assess participatory methods for engaging local stakeholders. The workshop included 22 participants from all sectors of society. Practical solutions to sanitation challenges were identified and later shared with a local community. Qualitative and quantitative analyses were used to assess impact and showed participatory methods were highly valued to encourage information sharing among widely varied stakeholders, and that video was a particularly successful approach when engaging with local communities. An 8-month follow-up survey of village members revealed excellent information recall, positive behaviour changes and a desire for future visits. Our evidence suggests that community-based participation helped identify solutions to WASH issues affecting rural communities in resource-poor settings. Engaging in a multicultural knowledge-share was particularly valuable as it enabled participants to recognize they have common challenges and allowed them to share low-cost solutions from their different communities. Our use of video was widely viewed as an ideal means of circulating findings, as it communicated information to people with a wide variety of community roles and to all age groups. Its relevance was increased by adopting a culturally appropriate context by involving local communities in workshop activities. We recommend that research in low- and middle-income countries should be mindful of the environmental context in which WASH is implemented, and encourage acceptance by engaging with communities through the use of varied participatory methods.

## INTRODUCTION

Worldwide, 2.4 billion people do not have access to improved sanitation facilities. Of these, an estimated 1 billion still practice open defecation (WHO and UNICEF, 2017). Poor sanitation and hygiene are some of the leading causes of morbidity and mortality in low- and middle-income countries (LMICs) globally, including diarrheal disease caused by cholera, dysentery and typhoid (Pruss-Unstun *et al.*, 2008). In 2017 it was estimated that 360 000 children under-5 years of age died due to diarrhea (WHO and UNICEF, 2017). As a result, Sustainable Development Goal 6.2 calls for access to adequate and equitable sanitation and hygiene for all and an end to open defecation (United Nations, 2018).

One of the key solutions to address these unacceptably high mortality rates is to encourage good hygiene and safe sanitation through the provision of improved sanitation facilities such as pit latrines with slabs and vents that prevent contact between excreta and community (Yimam *et al.*, 2014; WHO and UNICEF, 2017). However, a combination of population growth and slow progress means that access to improved sanitation facilities has decreased since 1990, particularly in rural communities (WHO and UNICEF, 2017). As a result, the practice of open-defecation increased from 204 to 220 million by 2017 (WHO and UNICEF, 2017).

Often the barrier to good hygiene and sanitation reflects a mix of ingrained cultural beliefs and socioeconomic constraints (Nakagiri *et al.*, 2015; Thys *et al.*, 2015; Wasonga *et al.*, 2016). Furthermore, many residents in LMICs do not receive education regarding the use or maintenance of pit latrines, even when facilities are made available (Grimason *et al.*, 2000). Calls have consequently been made for hygiene education programmes and promoting awareness of sanitation practices and latrine maintenance, but often take narrow vertical approaches and unsuccessfully try to target individual behaviour change (Grimason *et al.*, 2000; Garn *et al.*, 2017; O'Reilly *et al.*, 2017). Consequently, they often fail to acknowledge structural inequalities that shape the everyday lives of rural residents and lack relevance for local populations (O'Reilly *et al.*, 2017). As such, the views of the supposed beneficiaries of interventions are often hidden, and can be seen as having nothing to contribute or as being uncritically receptive (Biehl, 2016). A more inclusive approach is therefore needed to understand the complexities of dynamic community health systems, where knowledge and its application is employed in an appropriate sociocultural context and traditionally marginalized groups are sought and incorporated into projects as active participants (Leonardo, 2004).

To address this, it has been suggested that community-based participatory research (CBPR) approaches are used. CBPR is defined as a collaborative approach to research that equitably involves all partners in the research process and recognizes the unique strengths that each party brings (Minkler and Wallerstein, 2003). The process of CBPR begins with a research topic that is important to a target community and uses local knowledge and actions to improve health (Minkler and Wallerstein, 2003). Significantly, a participatory approach places emphasis on outside agencies joining with a community as equal partners in all parts of the research process and is seen to make research more responsive to community needs (Holkup *et al.*, 2004; Cargo *et al.*, 2007; Kamanda *et al.*, 2013; Brooks *et al.*, 2017).

Here we outline a CBPR approach that identified key water, sanitation and hygiene (WASH) issues in rural communities from Ghana and Uganda. This was achieved by engaging with a diverse range of participants to arrange a collaborative 3-day workshop and a subsequent community outreach visit. The impact of the approach on workshop participants was assessed immediately after the event and on the local community 8 months later.

## MATERIALS

### Aims and objectives

The main aim of this CBPR workshop was to improve WASH access and stewardship in a LMIC setting-based community with insufficient access to hygiene and sanitation infrastructure. Our activities encompassed six clearly defined objectives: (i) appraise the value of multinational knowledge exchange, (ii) examine the impact of engaging with varied stakeholders, including representatives from local communities, academia, non-governmental organizations (NGOs) and local government, (iii) assess the feasibility of using participatory methods to address sanitation challenges and inform the design of improved facilities, (iv) share workshop findings with a local rural community and (v) determine the longer-term impact of the approach.

### Context

The study was embedded as part of a wider study to, (i) assesses the diversity of pathogens in pit latrines using metagenomics approaches and, (ii) to conduct a WASH workshop to probe community level WASH challenges. To achieve our second goal, a multinational research team was selected from Ghana, Uganda, the UK and the USA. Both of the African partners face serious WASH challenges within their countries. In Uganda community members of slum settlements have poor knowledge of the link among water, sanitation, hygiene and health, as evidenced by the epidemics of cholera and typhoid, and a high incidence of diarrheal diseases particularly in children under 5 years of age (Musoke*et al.*, 2018). Similarly in Ghana, 18.75% of the population are reported as practicing open defecation (The World Bank, 2015), ranking it as the second highest sub-Saharan African country after Sudan for this practice (WHO and UNICEF, 2014). The UK and USA provided operational support for this part of the wider study. An established working relationship with Omni Med in Mukono, and academic staff from Makerere University and the University of Ghana, facilitated the engagement of a widely varied group of participants and enabled a visit to a Ugandan community; the demographics of this community was a suitable example of communities in the wider Mukono district. Omni med and the Mukono District Health Office have monthly meetings to discuss ongoing health initiatives and enabled our engagement with their Deputy District Health Officer (DDHO).

### Participants

The multinational partnership included village health team (VHT) members from Uganda (*n *=* *6), community environmental health workers (CEHWs) from the Ghanaian Community Water and Sanitation Agency (*n *=* *2), a Principle Investigator (PI) from the UK (*n *=* *1), academic co-investigators from the UK, Ghana and Uganda (*n *=* *1 × 3), PhD students from Uganda (*n *=* *4), a scientific manager from the UK (*n *=* *1), workshop facilitators from a USA–Ugandan NGO (*n *=* *2), and members of the Mukono NGO forum (*n *=* *2). A visit from the DDHO from Mukono, Uganda was arranged for the final day of the workshop.

VHTs are lay Ugandans acting in a voluntary capacity, who have been identified by their community as basic healthcare providers (Turinawe *et al.*, 2015). They are given training relating to major health programmes, so that they can mobilize communities to utilize formal health sector facilities (Turinawe *et al.*, 2015). They have a broad focus that includes maternal and child health, disease and sanitation and hygiene practices (Mays *et al.*, 2017). Unlike VHTs, CEHWs are employed by the Ghanaian Community Water and Sanitation Agency and report to the local government (Community Water and Sanitation Agency Ghana, 2018). This cadre has been in existence since 2009 and typically works in the communities from where they were selected, with a focus primarily on WASH issues. They have undertaken a 2-year training programme to acquire a certificate in Environmental Health and Sanitation and are able to issue legal notices to households who fail to adhere to sanitation and hygiene guidelines using powers granted to them by the Ghanaian legal system (Community Water and Sanitation Agency Ghana, 2018).

For the purpose of simplicity, in this study, VHTs and CEHWs will be referred to as community health workers (CHWs) from this point onwards.

### Workshop structure and content

A collaborative, multi-disciplinary, knowledge exchange workshop was organized. The workshop adopted a CBPR approach following recognized principles described by Israel *et al.* (Israel *et al.*, 2005), to understand, discuss and evaluate solutions to sanitation challenges of rural communities in LMIC settings. Two weeks prior to the workshop, a draft proposal for a 2-day workshop was circulated to all participants and the opportunity given to change the proposal to meet their needs. Emphasis was placed on the requirements of the Ghanaian and Ugandan CHWs who routinely work in rural communities. As CHWs felt more time was needed to discuss pit latrine construction and a tippy-tap (*an improvised hand washing source made out of a plastic bottle that can be refilled with clean water*) the workshop was extended to a 3-day event. The final workshop timetable can be found in Supplementary File S1. At the end of the workshop all attendees were awarded certificates of participation.

The total cost of the workshop was £6966 and included: International Travel £4500, Visas £155, Accommodation £822, Vaccinations & Medicines £322, Subsistence £752, Materials (stationary, printing, phone calls) £57, Computer Tablets £513. The computer tablets were gifted to the CHWs at the end of the project to enable them to continue presenting the workshop video to their respective communities.

### Location

The workshop was hosted by Omni Med, a USA-based, Ugandan-run NGO located in the Mukono district of Uganda (Omni Med, 2018). Mukono has a population of 600 000 people with a social structure comprised over 70% rural communities (Mukono District Local Government, 2015). More than two-thirds (69%) of households derive their livelihoods from subsistence farming (Uganda Bureau of Statistics, 2014). A recent WASH study in Mukono revealed that despite many households having access to pit latrines, the majority lacked improved sanitation facilities, such as hole-covers (84%) and hand washing facilities (70%) (Musoke *et al.*, 2018). The village chosen for our workshop visit was Kityabule, with a demographic distribution consisting of 860 individuals (497 Female, 363 Male) with 49% under 16 years of age and 5% over 65 years of age.

Omni Med were chosen as they have trained and maintained 1250 CHWs since 2008 across the Mukono district and have strong links with village leaders and local communities. The location was also in close proximity (∼25km) to Kampala’s major transport links, providing easy access for international participants.

### Participatory visual methods

Interactive methods and participatory visual methods (PVMs) were used to illustrate key topics, including photography, video, feature mapping, drawing and practical demonstrations (Richards, 2011). Such methods encourage participants to document findings, reflect on their personal experiences and promote dialogue (Wang and Burris, 1997). They are used to focus the research priorities and promote social change by aligning them to local needs, and only require minimal literacy (Hergenrather *et al.*, 2009). For this reason they have traditionally been used with marginalized and under-represented groups, and have been credited with helping to shift research into communities (Lipson, 2017). Drawing methods are thought to be particularly powerful in resource-poor environments, since they can be implemented easily and inexpensively, do not require literacy, and can also help to overcome language barriers where multinational partnerships are involved (Literat, 2013). Below we describe each of the participatory methods used in our study.

#### Participatory photography and video

A Ugandan CHW volunteered to capture photographic images and video throughout the course of the workshop, and was given an information sheet and 3 h of camera training (Supplementary File S2); to capture topics that the CHW felt important. No restrictions or limits were suggested. Informed consent was obtained prior to the workshop and an hour-long interactive session was used to discuss the importance of informed consent. Photos and videos were later used to compile a short educational video to convey workshop findings to rural communities The length and content of the video was planned by all members of the workshop and followed by a round-table review to produce a culturally sensitive version for presentation to communities (Elimu Health, 2018). The video was created at the end on the final day of the workshop and was focused on hand washing and pit latrine construction. Two versions were made with commentary in either Ugandan or Ghanaian dialects, and popular local music was added to increase the cultural relevance. The video was made using iMovie version 10.1.8 and the editing process demonstrated to the Ugandan and Ghanaian CHWs for ease of replication in future self-sustained workshops.

#### Feature mapping

Workshop participants were split into groups of four-five people and asked to draw one of their communities, placing attention on WASH features such as unsecured water sources, protected water sources (PWSs), drainage and latrines. Completed maps were presented to all workshop members by an elected head of the group and round-table discussions used to share cross-cultural perspectives (Van den Bossche *et al.*, 2006; Perkins, 2007; Iqbal *et al.*, 2016; Saunders *et al.*, 2016). This exercise took 60 min.

#### Participatory drawing

Participants working in groups of four-five people were asked to draw and annotate an ideal design for a pit latrine. Group heads were elected to present the findings after which there were round-table discussions. This exercise lasted 60 min.

#### Practical demonstrations

A basic hand washing approach (tippy-tap) was demonstrated by Ugandan CHWs. This exercise lasted for 90 min and video footage was included in the final workshop video.

### Workshop evaluation

Handwritten field notes were taken by the study PI, distributed to two workshop facilitators for validation, and a permanent record created at the end of each of the 3 days. At the end of the workshop a questionnaire was distributed to participants to capture their appraisal of the workshop content (Supplementary File S3). The questionnaire was anonymous to encourage honest feedback.

### Month 8 community follow-up assessment

Eight months after the community outreach, Ugandan workshop CHWs and a workshop facilitator returned to the community and surveyed community members who were available and had attended the original visit, to assess the longer-term impact of the workshop (Supplementary File S3). All interviews were conducted via translators in the local language.

### A transformative framework

A transformative framework (TF) was first described by Mertens (Mertens, 2003). It was later modified by Sweetman *et al.* (Sweetman *et al.*, 2010) and included 10 criteria for evaluating studies such as ours. We used this method as an evaluation tool for the current study since our research used a mixed-methods approach and concerned WASH issues affecting rural communities. The method is comprehensive, widely regarded as a means of assessing the inclusion of advocacy in mixed methods, and takes into account the individual’s worldview and value assumptions.

### Ethical approval

Research Ethics Committee approval was obtained from the Mengo Hospital Research Ethics Committee (79/07-17) and the Uganda National Council for Science and Technology (NS-613). Approval was also granted from The Department of Education Research and Ethics Committee (DREC) at the University of Oxford (ED-CIA-18-149). The research conformed to the principles embodied in the Declaration of Helsinki. All participants signed informed consent forms which had been approved by the DREC. As dictated by local custom, verbal consent was obtained from the village leader prior to conducting the community outreach workshop on the final day. All persons who appeared in photos or videos were adults over the age of 18 and gave informed consent. In the event of controversial images being captured, any major issues of concern would be referred to Omni Med staff since they were in the best position as local health advocates to manage such issues in a culturally appropriate manner. Issues surround the ethics of photovoice have been discussed in the literature in detail in the past (Creighton *et al.*, 2018).

### Patient and public involvement statement

All workshop participants were actively involved in workshop design and implementation with particular emphasis placed on the needs of the CHWs. Local NGO members coordinated with a local village leader, village elders and CHWs with the aim of presenting workshop findings to their community. Permission was granted and community members were encouraged by local representatives to attend the education event. Group discussions about WASH issues were discussed with the community via translators, the workshop video was presented (in a local dialect) and a tour of village water sources and latrines was arranged. Patients were not involved in the study.

## RESULTS

### Cross-cultural knowledge exchange and a range of participatory approaches ensures that all stakeholders win

Following completion of the workshop a questionnaire was completed by all 21 workshop participants (Supplementary File S5). It included 11 questions, with Q1–7 establishing whether the participatory methods were considered valuable, and Q8–10 probing whether the approaches could be further improved. Q11 was an open question requesting any other comments. To provide a summary of the responses, the first 10 questions were enumerated as either positive or negative responses (Figure 3A). In this section we summarize all of the responses, and in later sections provide a more detailed analysis of each participatory method.

The majority of respondents reported that the workshop had been valuable. Several key areas were identified as source of value, with positive themes focusing on the value of a community-based study, the broad diversity of participants, the multinational nature of the workshop and the use of photography and video making (Q1–3). One participant commented that ‘The workshop was really participatory and exchange of information from various people with different backgrounds’. The majority of respondents (19/21) also reported that they had been given opportunities to actively participate (Q4–6), with qualitative responses focusing on participant diversity as a novel approach, stating that ‘Sharing knowledge between people on ground + researcher + policy maker. Unique way of sharing + disseminating info’. The cross-cultural knowledge exchanges were similarly seen as a strength, with one participant highlighting ‘that everyone faces the same challenges’. Most participants (16/21) felt that they had not experienced difficulties in engaging with the workshop, while the others (5/21) did not feel there were any major barriers to overcome, and there was no common theme to suggest a single challenge to address. Areas for improvement included accessibility to the remote location and the ability of participants to understand everything that was presented to them. Some challenges were also highlighted in Q2 and 3, which had lower scores than for other questions as they specifically queried participants’ dislikes and their suggestions for improvement (Figure 3A). Their key suggestions for improving the workshop included extending invitations to other key community members such as religious leaders, and that the workshops should be longer and implemented more often.

When participants were queried about the suitability of the approaches, the participatory mapping exercise (Q8), drawing exercise (Q9) and photos/video (Q10) were all highly valued as means of engaging communities (Figure 3A). An open request for comments (Q11) revealed that the workshop was considered ‘interesting and engaging’, ‘everyone was equal’ and that it should be extended to other communities. It was again also suggested by another participant that village leaders, the church and other communities should be involved in the workshop.

### Feature mapping promotes equality among participants and helps identify challenges and solutions that are shared by distinct communities

**Fig. 1:**
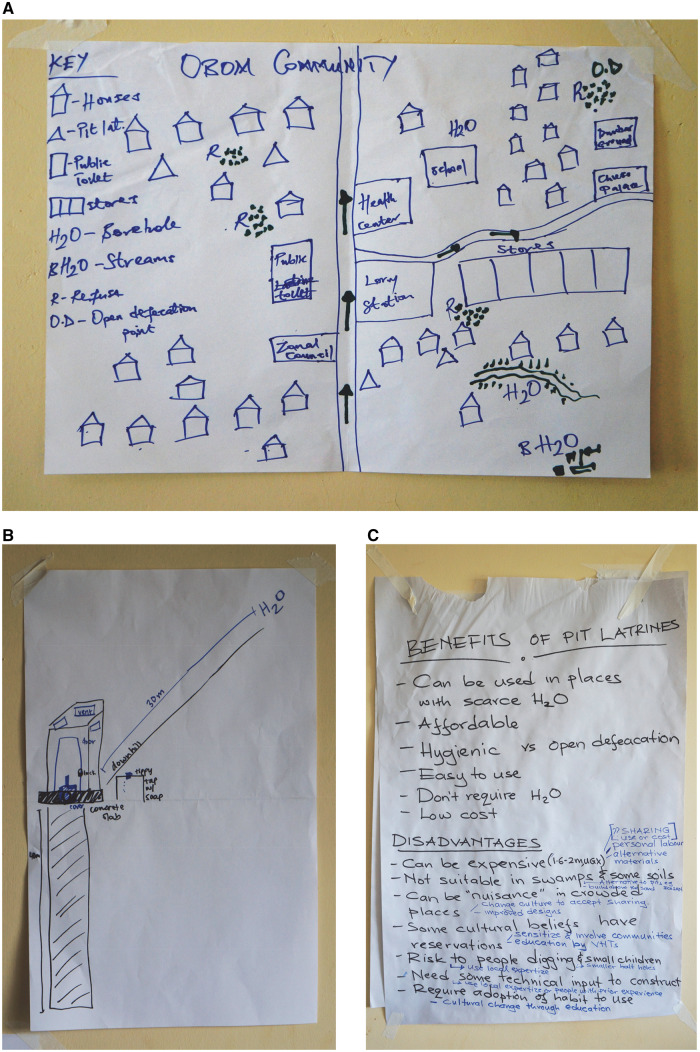
Examples of feature mapping (**A**), participatory drawing (**B**) and conclusions from round-table discussions about pit latrines (**C**). The map was drawn by a Ugandan CHW, demonstrating some of the key water and sanitation facilities in their local area. The participatory drawing was created by a Ghanaian CHW and describes some of the key features of pit latrine construction. Original drawings in (**A**) and (**B**) used black pen and altered using blue pen during group discussions. These discussions resulted in the advantages and disadvantages that are seen in (**C**) and summarized in Table 1.

Mapping key WASH features identified common challenges faced by communities in Ghana and Uganda, and allowed workshop participants to discuss potential solutions to these issues (Figure 1A). Included in the maps were the number and locations of houses, shops, natural springs, bore holes, streams, refuse, pit latrines and public toilets within their communities. Themes included the challenges of public use, water contamination and open defecation practices in these areas and their negative impact on the surrounding people and facilities.

The workshop questionnaire showed that participants valued the interactive nature of the exercise, with CHWs in particular, feeling that map drawing was a valuable method of conveying important issues in their local areas (Figure 3A, Q8). They acknowledged that by partnering with participants from different countries, ‘this got us talking and learning about each other’ and ‘made everyone understand how the contamination can occur’. Other participants commented on how mapping ‘illustrates communities in relation to water, sanitation and hygiene’ and how it can be used as a means of providing ‘an overview on how individual communities could trace disease’ (McLeod, 2000). When participants were also invited to indicate what they liked or disliked (Q8), five participants did not answer the question or expressed reservations, with one addressable suggestion being that prior preparation could enable ‘more realistic assessments’ of feature maps.

### Participatory drawing facilitates discussions about specific difficulties that could be missed if local stakeholders do not feel motivated

Drawing the design for an ideal pit latrine focused on the need for a robust construction, stable concrete slab, hole-cover, door, vent pipe, hand washing facilities and a location downhill from water sources (Figure 1B). In particular, CHWs found the exercise beneficial for learning about the varied construction methods and materials in different countries. A discussion around whether a lock should be used on pit latrines received considerable debate and revealed that the majority of Ugandan and Ghanaian participants (15/18) felt a lock should be used if the latrine was their property, citing reasons such as avoiding damage to the latrine and hole-covers being stolen. The remaining Ugandan and Ghanaian participants (3/18) felt that by using a lock, other community members may choose to defecate in the open if they did not have access to the key.

A wider discussion around the general advantages and disadvantages of pit latrines was facilitated by a Ugandan PhD student and several themes emerged (Figure 1C). These included affordability, their use in places with water-scarcity, improved hygiene compared to open defecation and ease of use. Several barriers to the construction and use of pit latrines, as well as corresponding solutions, were also identified and are shown in Table 1.

**Table 1:** Advantages and disadvantages of pit latrines identified during round-table discussions

Challenges/disadvantages	Potential solutions
Expense of construction	Using lower cost materials Providing labour if another organization provides the materials Potentially sharing use and/or cost
Challenges of construction in swampy environments or areas with poor soil quality	Reinforcements to be used in the linings to stop pit walls from breeching e.g. old lorry tyres Alternatives to pits e.g. Ecosan toilets built above the soil have been used in areas where the water table is high
Perception that pit latrines are a nuisance in crowded places	Changing cultural attitudes around sharing Improved designs working alongside those who will be using the latrines
Cultural stigmas and beliefs around the use of pit latrines	Educate communities around the use of pit latrines by working with village leaders Work with CHWs to conduct community-based training
Risk to those constructing pit latrines e.g. risk of pit wall collapse	Dig shallower holes Use local construction-expertise Reinforcements to be used in the linings to stop pit walls from breeching e.g. old lorry tyres
To construct a good pit latrine some expertise and technical input is needed which is not always available	Work with CHWs or people who have prior expertise in construction Hold community-based education workshops on how to safely construct a pit latrine

The workshop questionnaire revealed that these discussions were valued by 19/21 respondents, with a further two not answering the question (Figure 3A, Q9). It was generally noted that the method was ‘simple and clear’, ‘different communities/nationalities had varied answers to the challenges’ and that it helped participants ‘to think about key design challenges in resource poor environments’. None of the participants suggested any major changes to this approach if another workshop was conducted.

### Photography and video-making eases integration and direct engagement with communities

**Fig. 2:**
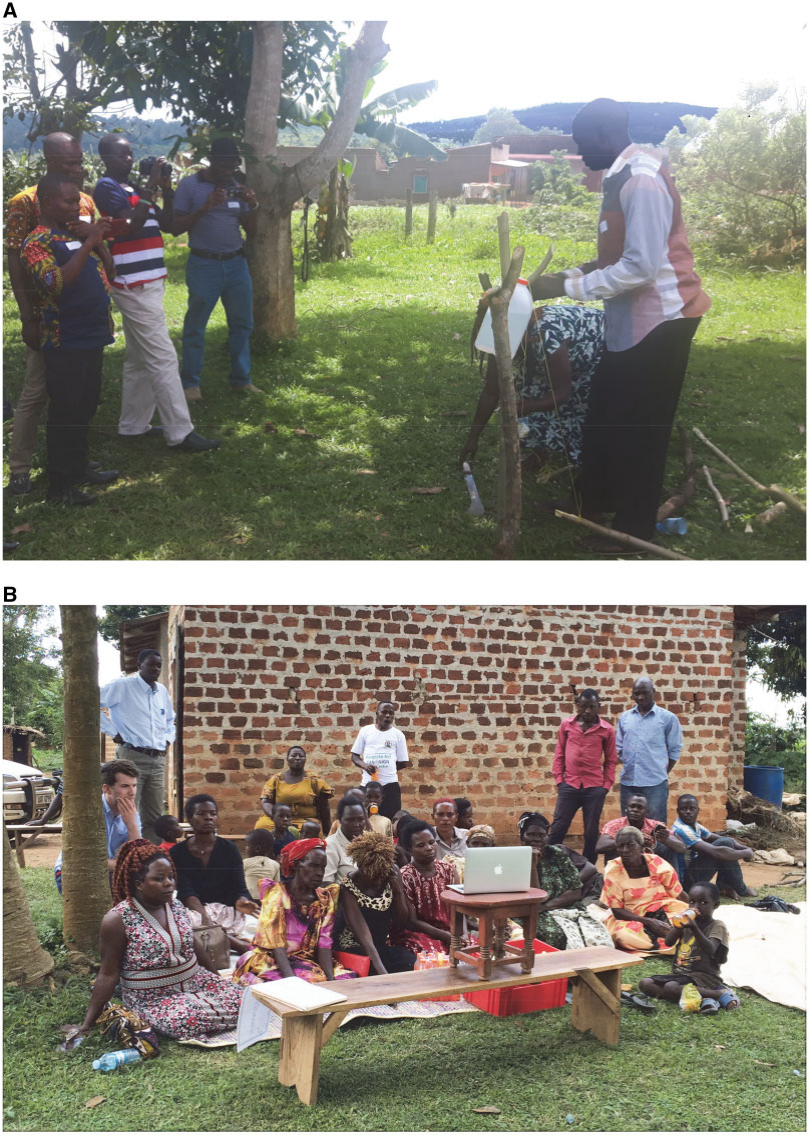
Two Ugandan CHWs demonstrating how to construct a low-cost tippy-tap for hand washing (**A**). In the background Ugandan and Ghanaian CHWs can be seen videoing the demonstration. In (**B**) villagers watch the workshop video (05:39 min; Elimu Health 2018).

By encouraging CHWs to photograph the workshop we were more readily able to identify the themes that were considered important to them and incorporate these into a subsequent video that was presented to a local community (Figure 2A). Feedback from a Ugandan academic researcher indicated that this was a good way of involving CHWs in the research process, stating ‘I really liked seeing the CHWs in this workshop using the cameras to document the process. I have never seen another Ugandan being the person who is the one documenting what is occurring. It helps the world to see things through our eyes’*.* One CHW commented that ‘Videos attract the attention of village folks’, while another *c*ommented that the workshop had taught them that the use of photographs and videos could be used as a learning and community engagement tool since ‘they attract attention’. This was in contrast to the Ghanaian practice of photographing poor sanitation practices and using this as documentary evidence in court. They commented that using photography to promote positive outcomes represented a shift away from visual images as a deterrent, and towards an educational role.

**Fig. 3:**
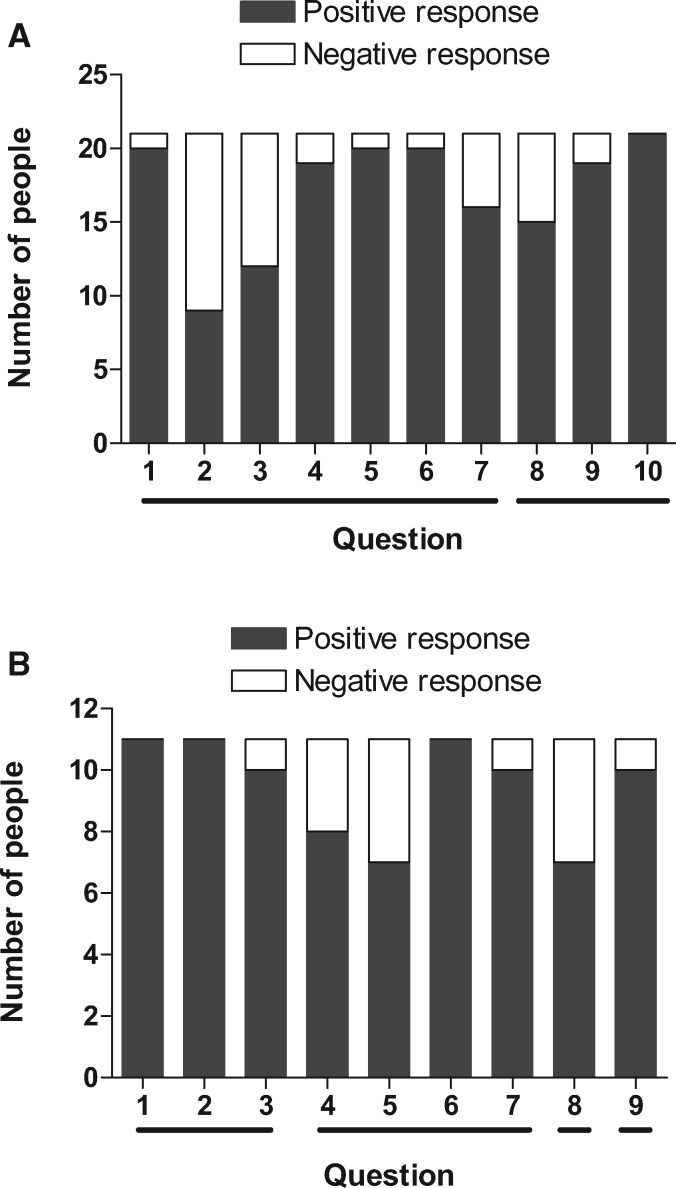
A graphical representation of responses to questionnaires. (**A**) The workshop questionnaire was completed by all participants (*n *=* *21) and consisted of 11 questions, the first 10 of which were enumerated and are shown here. The horizontal bars on the *x*-axis show thematic groups described in the text; the first theme probed the value of participatory methods, and the second theme how the methods could be improved. A more detailed description of the questionnaire, answers and scoring can be found in Supplementary Files S3 and S5, respectively. (**B**) A graphical representation of responses (*n *=* *11) from an 8-month follow-up questionnaire within the local community that was visited during the workshop. Of the 10 questions, 9 were enumerated and are shown here. The horizontal bars on the *x*-axis show thematic groups described in the text; from left to right the themes established whether the visit was remembered, advice would be recalled, there was positive behaviour change and whether improvements could be made. A more detailed description of the questionnaire, answers and scoring can be found in Supplementary Files S4 and S6, respectively.

Feedback from the questionnaire revealed unanimous support for the use of photos or video (Figure 3A, Q10). Participants expressed that ‘visual aids are the most effective means of communication’ and that ‘visual media is more engaging than just audio’. Importantly, ‘photos are long lasting and children in particular can help spread the information’ as ‘they really love to share with others what they saw and also want to practice it’. It was also noted that ‘smart phones are now common in even rural settings and videos adapted to this platform are a great way of reaching the masses with important messages’ (CIPESA, 2018; Kadiyala *et al.*, 2018). The only suggestion for improving video was the addition of subtitles (in response to Q3).

### Cross-cultural participation and the involvement of local communities facilitates sustainable WASH practice and encourages knowledge exchange

Construction of a tippy-tap was demonstrated by a local community member, which led to discussions between Ghanaian and Ugandan CHWs on the importance of educating local community members to use soap when washing their hands. All CHWs were in agreement that local community members should be expected to purchase their own soap, rather than rely on gifts from local NGOs. One Ugandan CHW justified this by drawing parallels with a government led initiative to distribute free mosquito nets and a resultant feeling that communities lacked ownership and were therefore unwilling to pay for net repairs and they rather used the nets to fish or protect their crops. It was consequently agreed that if community members were unable to afford soap, CHWs should hold outreach-training sessions where community members could be taught how to make it.

### Reaching out to communities with culturally sensitive information is essential for encouraging positive behaviour changes

A community outreach visit was organized for the final day of the workshop where the workshop video was presented to 17 village members consisting of adults and children (05:39 min; Elimu Health, 2018).

Following the video presentation, villagers were asked to provide their immediate opinions. They responded (via local translators) by stating that ‘It is so good to see this. When you see this, you remember more. It will help us’*.* Other village members commented that they enjoyed seeing a local CHW in the video documenting safe sanitation practices and correct hand hygiene techniques and enjoyed our use of familiar local music. Another member of the village commented that ‘The latrine you showed in the video had concrete walls and concrete slabs and a light bulb. We cannot afford that in the village so what do we do?’ In response, Ugandan and Ghanaian workshop CHWs explained that different materials could be used to construct pit latrines. Villagers later asked for a second viewing of the video.

Following the second video screening, workshop members were invited by CHWs to view PWSs and pit latrines within the village. Challenges associated with these were discussed at length by the villagers and workshop members. For example, one PWS was experiencing a diverted-flow. It was observed that people had walked on the adjoining land which had compacted the soil and resulted in water no-longer moving through its natural underground channel. Solutions were discussed by villagers and both Ugandan and Ghanaian CHWs and it was concluded that fencing off the immediately surrounding land may alleviate the problem. Pit latrines in the village were well maintained with high-standards of sanitation, but experience of similar latrines in their own communities enabled Ghanaian CHWs to suggest low-cost improvements, such as plywood hole-covers and repairing holes in the latrine walls with clay. These examples highlighted the benefits of cross-cultural knowledge exchanges when applied directly to village communities.

### Community-led video creation is ideal for sustainably guiding education programmes

To assess our longer-term impact, a member of the USA–Ugandan NGO returned to the village 8 months later. Seventeen village elders attended the initial visit, and of these 11 were available for interview during the follow-up. Villagers were asked 10 questions (Supplementary File S4). Q1–3 established whether the visit was remembered and its purpose, Q4–7 probed whether details of the advice could be recalled, Q8 whether this had resulted in a positive behaviour change, and Q9 and 10 whether the visits could be improved. The first nine of these questions were enumerated as either positive or negative responses according to criteria described in Supplementary File S6. Figure 3B summarizes this analysis and shows that the majority of villager interviewed remembered the visit (Q1 and 2), and that most could recall the video information that was provided (Q3). Clear descriptions of specific details within the main themes (latrines/hand washing) were less clearly recalled (Q4 and 5), but general themes around the use of soap, water and hole covers were better remembered (Q6 and 7). For all but one villager, at least one positive change in latrine use or hand washing was noted as a consequence of the visit (Q8), with seven villagers describing two or more changes. When asked what was most memorable, ten villagers clearly described at least one of the events that happened during the visit, all of which were included in the contents of the workshop video (Q9). Q10 probed how future visits could be improved. It was agreed by all respondents that future visits would be welcomed and should include additional topics, practical help in constructing latrines and renovating water sources, and the provision of hole-covers and doors. The full answers to these questions are shown in Supplementary File S6.

### Appropriate evaluation of community-based projects is a must to for the satisfactory meeting of local needs

In addition to the 8-month follow-up, a global evaluation of our study was also undertaken according to a series of criteria used to assess mixed-methods studies, and described by the TF of Sweetman *et al.* (Sweetman *et al.*, 2010). This showed that that our study met all 10 criteria of this framework, the evaluation of which is shown in Table 2.

**Table 2:** A TF mapped to elements of our study

TF criteria	How our study addressed the criteria
Authors openly reference a problem in a community of concern	We recognize that improved sanitation facilities are often lacking in rural areas of LMICs We identify that low-cost, contextually appropriate solutions need to be found to encourage usage and continued maintenance We ensure cultural relevance by engaging community-based groups from Ghana and Uganda
Authors openly declare a theoretical lens	Our work addresses critical theory and global health as described in the introduction of this manuscript
Research questions written with an advocacy stance	Our research demonstrates the importance of engagement with local stakeholders to identify issues they consider important
Literature reviews include discussions of diversity and oppression	We acknowledge that those in rural areas of LMICs are more likely to face barriers to improved WASH facilities We engaged stakeholders at all levels of the community and have engaged both male and female participants throughout our study
Authors discuss appropriate labelling of the participants	We consider and refer to participants as partners in the research proposal Roles are referred to only where they provide a useful context and are excluded from anonymized responses to questionnaires
Data collection and outcomes benefit the community	CHWs worked together to share ideas that they will use in their future practices Ideas that emerged from the workshop were translated into a video format and shared with the community to address WASH challenges Videos were developed in both Ghanaian and Ugandan dialects, and with appropriate local music, to increase their cultural appeal in different communities
Participants initiated the research, and/or were they actively engaged in the project	All participants were actively engaged with the project design, implementation and evaluation An iterative approach was used to enable us to fulfil all participants needs The use of participatory methods ensured all workshop participants were actively engaged throughout the 3 days
Results elucidate power relationships	From design through to implementation, we have given all participants equal stakes in identifying workshop themes We acknowledge that simply involving a policy maker in the collaborative workshop will not necessarily translate into policy changes, but hope that by this will be more likely
Results facilitate social change	We specifically reached out to local community members in a rural village, and CHWs from Ghana and Uganda were able to make practical suggestions for improvement We shared the findings of the workshop with local communities using a culturally relevant video created by workshop participants Our 8-month assessment suggests that the visit has had a longer-term impact with positive changes in sanitation practices
The authors explicitly state the use of a TF	We have explicitly used a 10-point assessment using the TF as described by Sweetman *et al.* (Sweetman *et al.*, 2010)

## DISCUSSION

Here we describe a mixed-methods research project that addresses key WASH challenges in LMICs to address the need for improved sanitation and hygiene education. To assess the use of participatory methods, a 3-day workshop was organized and run in partnership with community representatives from Ghana and Uganda. Feedback from this workshop revealed that the use of a hands-on, cross-cultural approach that connected a wide variety of stakeholders was seen as highly valuable. Using the TF of Sweetman *et al.* (Sweetman *et al.*, 2010) to assess the study, we showed our work addressed the needs of local stakeholders, a conclusion that was supported by the extensive level of information recall by village members at the follow-up visit.

In the past, partnerships between researchers and society have often been unbalanced, with researchers being perceived as owning ideas, and communities not directing the research (George *et al.*, 2015). Questionnaires showed that the success of the current workshop arose from our partnership with community members to organize the event, the diversity of multinational participants, and our use of a participatory-based approach for the workshop design, implementation and community outreach. Using a combination of drawing, photography, practical demonstration and video, as opposed to relying on a single approach, provided a wider range of opportunities to involve multiple stakeholders, and the benefits we identified were consistent with findings of George (George *et al.*, 2018). Other participatory methods have also been applied when working alongside stakeholders with varied cultural and contextual backgrounds, with different communication styles, approaches to completing tasks and alternative epistemologies being reported (Kiss, 2005; George *et al.*, 2018). Here, we found that all of the methods were valued for promoting open workshop discussions as was the cross-cultural learning experience. By encouraging the CHWs from the different communities to lead these discussions we were able break down usual hierarchies and make discussions focused on wider community needs. We found that video was considered particularly useful for engaging with community groups and the high level of recall by village members at our 8-month follow-up supports this view. However, it should be noted that PVMs are not without limitation as they require the need for facilitators who are familiar with the techniques (O'Donovan *et al.*, 2019).

Beran *et al.* (Beran *et al.*, 2017) has noted that those working and living in LMICs are better placed to define issues of importance than people living thousands of miles away in high-income countries. In our own study, a round-table discussion identified that the use of pit latrines presents challenges that are complex and linked to cultural factors. For example, we found that many people were reluctant to invest in building their own private pit latrines since there were uncertainties regarding land ownership, a finding that has been noted elsewhere (Awunyo-Akaba *et al.*, 2016). Solutions to these challenges largely came from our CHWs who are aware of the sociocultural sensitivities, economic constraints and logistical challenges in resource-poor environments. This suggests that policy makers and programme managers should make efforts to understand what shapes people’s motivations, and work with local stakeholders to develop realistic solutions (Awunyo-Akaba *et al.*, 2016). Working closely with government has been demonstrated as a powerful means of achieving this, and in Rwanda is responsible for pit latrine coverage now standing at 82.2% (Ekane *et al.*, 2014; Nakagiri *et al.*, 2015). In our study, participatory approaches were used to promote interactions between CHWs, researchers and local government representatives. Based on the findings of our questionnaires, all of these groups felt that this approach encouraged participants to think beyond the logistical issues that prohibit adoption of safe sanitation and good hygiene practices, and to also consider sociocultural barriers that can be deeply rooted in communities. One example of this was provided by a Ugandan CHW, who described the locally held belief that pregnant women may miscarriage if they use a pit latrine. Such thinking can be missed without the open discussions offered by a participatory approach, and yet they are important when implementing improved sanitation since they are likely to play a major role in their overall adoption (Dube and January, 2012; Thys *et al.*, 2015). We therefore advocate that community-based research should be considered early in a work plan and should incorporate participatory methods to encourage dialogue from stakeholders at all levels i.e. the targeted community should feel the real ownership of the training programme. Although it is already recognized that sociocultural factors are critical to the medical encounter, unfortunately such practices are often omitted from undergraduate, graduate and continued health education (Zweifler and Gonzalez, 1998; Culhane-Pera *et al.*, 2000).

A potential limitation of a study such as this is the cost associated with conducting a knowledge exchange between varied stakeholders from different countries. However, we consider this valuable as it ensures that local contexts inform the wider project goals and exposes all project members to wider global health considerations. This opinion is supported by the high value that participants placed upon the workshop and by the longer-term impact of our approach upon the local village community. In our study, the full costs (£6966; see materials for a breakdown of these) of the workshop were included in our initial grant proposal, and we believe that as many funding bodies are keen to promote research beyond traditional scientific boundaries, these costs should always be included where possible. In addition, it should be noted that the initial costs of our workshop were high, due to several international partners having to convene in one location. In comparison, material costs were low (£57), and other alternative collaborative approaches such as virtual meetings would significantly reduce overall expenditure. A second challenge was the logistical implementation and coordination of a multinational workshop. To address this, we ensured passports and necessary travel documents were acquired well in advance, and regularly corresponded to the strategic coordinator of Omni Med and the team from Ghana to ensure we had the necessary approval letters and travel documentation. Finally, it should also be noted that involving individuals more fully in the research process can also raise ethical issues (Black *et al.*, 2018). To address this challenge we ensured that informed consent was obtained for our photography and held an hour-long interactive session where the importance of informed consent around the use of photography and video was discussed.

In summary, our workshop engaged varied stakeholders from within academic research, policy, practice and civil society. While it is important to note that we do not claim this approach will necessarily bring about changes in policy, we believe that the engagement of these different groups starts a discussion and makes it more likely. A major success of our study was the adoption of community-based participation, which helped ensure a culturally sensitive knowledge exchange that captured a diverse range of views. We believe that such an approach is an important step towards challenging the research-led approach sometimes taken within the field of global health. It also encourages those working in community settings to consider approaches that will engage community members, and to seek contextually appropriate solutions (George *et al.*, 2018). CHWs represent an existing and valuable community resource through which the training can be channelled. The use of photography and video was particularly valued by both workshop participants and the village community, and the use of local dialects and music increased its cultural-sensitivity and improved audience engagement. The impact of such an approach was indicated by the high level of information recall and behaviour changes that were documented 8 months later. We encourage the engagement of local stakeholders in the planning and execution of community projects and advocate the use of participatory methods to promote this.

## SUPPLEMENTARY MATERIAL

Supplementary material is available at *Health Promotion International* online.

## AUTHORS’ CONTRIBUTIONS

All the authors have made a substantial contribution to (i) the conception and design and/or the analysis and interpretation of data, (ii) drafting the article or revising it critically for intellectual content and (iii) all authors approve the submitted version.

## Supplementary Material

daz127_Supplementary_Data
