# Supplementary material for: Participatory approaches, local stakeholders and cultural relevance facilitate an impactful community-based project in Uganda
Source: Health Promot Int. 2020 Feb 18;35(6):1353–68. doi: 10.1093/heapro/daz127 (PMC7785315; doi:10.1093/heapro/daz127)
Supplement: daz127_Supplementary_Data [file daz127_supplementary_data.zip › SuppleFile1RP.docx]

**Supplementary File 1:**

| **Workshop – Day 1** | | | |
| --- | --- | --- | --- |
| **Time** | | **Activity** | |
| 9.30am | | Welcome and icebreaker | |
| 10:00am-11.30am | | Demonstration of how to use cameras and videos | |
| 11.30am-11.45am | | Tea and biscuits | |
| 11.45am-12.30pm | | Practice using camera and videos to take photos and videos | |
| 12.30pm-13.15pm | | Lunch | |
| 13.15pm-15:00pm | | Demonstration of tippy tap construction by Ugandan CHWs, and group discussion around informed consent when taking photos and videos | |
| 15:00pm-15.30pm | | Closing of day one. Opportunity to ask questions and share ideas. | |
|  |  |  |  |
| **Workshop – Day 2** | | | |
| **Time** | | | **Activity** |
| 9.30am | | | Welcome, and discussions covering any questions or concerns from day one |
| 10:00am-11.30am | | | Participatory mapping workshop |
| 11.30am-13:00pm | | | Presentation on pit latrines by Dr. James O’Donovan (Univerisity of Oxford), and a participatory drawing workshop |
| 13:00pm-14:00pm | | | Lunch |
| 14:00pm-15.30pm | | | Group discussions between CHWs and researchers on improved sanitation and hygiene strategies  Group planning of an educational video  Filming of key sections for video  Editing of video |

| **Workshop – Day 3** | |
| --- | --- |
| **Time** | **Activity** |
| 10:00am | Welcome and covering any questions or concerns from Day One and Two |
| 10:00am-10.30am | Presentation from Isaac Ddumba, District Health Officer of Mukono |
| 10.30am-12:00pm | Presentation of photos, pit latrine drawings, maps and presentation from Dr. Ankur Mutreja on ‘Metagenomics and research findings’  Presentation of draft video and feedback. Further editing of video following feedback  Presentation of certificates and t-shirts to delegates |
| 12:00pm-14:00pm | Lunch |
| 14:00pm-16.30pm | Visit protected water sources, pit latrines in local village, and CHWs to share the video and ideas with local villagers in a knowledge-exchange exercise |
| 17:00pm-19.30pm | Closing of Workshop and final dinner |
