# Supplementary material for: Participatory approaches, local stakeholders and cultural relevance facilitate an impactful community-based project in Uganda
Source: Health Promot Int. 2020 Feb 18;35(6):1353–68. doi: 10.1093/heapro/daz127 (PMC7785315; doi:10.1093/heapro/daz127)
Supplement: daz127_Supplementary_Data [file daz127_supplementary_data.zip › Supplementary File 2[2].docx]

**Supplementary File 2:**

**Taking photos and videos – a few suggestions**

**Photos**

- To make sure the photo quality is good, try practice taking a few photos. This will help you ensure the picture is clear.
- If you need to, adjust the settings on the camera.
- Try to find a place with good lighting, that isn’t too dark or bright, before taking your photo.

**Video**

- Practice taking different videos with the camera, to make sure the lighting quality and sound quality are good.
- Avoid places that are very noisy, that will make it difficult to hear what you are saying. For example, avoid taking the video next to a busy road or where there are children playing. Try to find somewhere quiet.
- Try to keep the camera still when you are taking the video. You can do this by using the tripod. If you don’t have a tripod you could place the camera on something else.

- Try look directly into the lens of the camera if you are talking to an audience – this way the final video will look much better!
- Remember, if you are not happy with the video you can delete it and try again!
- Most of all, have fun making the pictures and video, and feel free to express your views in any way you wish.
