# Supplementary material for: Participatory approaches, local stakeholders and cultural relevance facilitate an impactful community-based project in Uganda
Source: Health Promot Int. 2020 Feb 18;35(6):1353–68. doi: 10.1093/heapro/daz127 (PMC7785315; doi:10.1093/heapro/daz127)
Supplement: daz127_Supplementary_Data [file daz127_supplementary_data.zip › Supplementary File 3[2].docx]

# **Supplementary File 3:**

# **Workshop Evaluation: to be completed by workshop participants**

# **I identify as: (community health worker) (researcher) (facilitator) (other: explain)**

*PURPOSE: Were the participatory methods helpful*

**1. What did you enjoy about the workshop?**

**2. What did you not like about the workshop?**

**3. How do you think things could have been improved?**

**4. Did you feel involved in the workshop?**

**5. Were you given v to participate in the workshop?**

**6. What did you learn from taking part in the cross-cultural exchange between Uganda-Ghana-UK-USA?**

**7. Did you face any difficulties? If yes, what were they?**

*PURPOSE: Could the methods be improved*

**8. What did you like/dislike about the mapping exercise?**

**9. What did you like/dislike about the pit latrine drawing exercise?**

**10. Do you think using photos or video could be useful for community engagement? If yes, why?**

**11. Please write any other comments you wish to make not covered by the above questions here:**
