# Supplementary material for: Participatory approaches, local stakeholders and cultural relevance facilitate an impactful community-based project in Uganda
Source: Health Promot Int. 2020 Feb 18;35(6):1353–68. doi: 10.1093/heapro/daz127 (PMC7785315; doi:10.1093/heapro/daz127)
Supplement: daz127_Supplementary_Data [file daz127_supplementary_data.zip › Supplementary File 4[2].docx]

**Supplementary File 4:**

# **Eight Month Assessment: to be completed by an official representative**

# *PURPOSE: Do village members remember the visit and its purpose*

**1. Do you remember visitors coming to your community? (circle one)**  **YES NO**

**Notes:**

**2. Do you remember why the visitors came/what they talked about? (circle one)** **YES NO**

**Notes:**

**3. Please share with us what you remember about what the visitors told you?**

*PURPOSE: Do village members remember details from the visit*

**4. What do you think was the most important information that was shared with you?**

​

**5. How long should you wash your hands?**

**6. What are some strategies you can use to encourage hand washing?**

**7. Can you identify any features that can improve or are important in pit latrines?**

*PURPOSE: Has the visit had an impact*

**8. Have you made any changes to your habits since the visitors came?**

**Notes:**

*PURPOSE: Is there potential for improvement*

**9. What features about the visit stuck out to you or do you remember most?**

**10. What do you think should be done differently for visits in the future?**
